# *In Vivo* Deficiency of Both C/EBPβ and C/EBPε Results in Highly Defective Myeloid Differentiation and Lack of Cytokine Response

**DOI:** 10.1371/journal.pone.0015419

**Published:** 2010-11-03

**Authors:** Tadayuki Akagi, Nils H. Thoennissen, Ann George, Gay Crooks, Jee Hoon Song, Ryoko Okamoto, Daniel Nowak, Adrian F. Gombart, H. Phillip Koeffler

**Affiliations:** 1 Division of Hematology and Oncology, Cedars-Sinai Medical Center, University of California Los Angeles School of Medicine, Los Angeles, California, United States of America; 2 Division of Research Immunology/BMT, Children's Hospital Los Angeles, Los Angeles, California, United States of America; 3 Department of Pathology and Laboratory Medicine, David Geffen School of Medicine, University of California Los Angeles, Los Angeles, California, United States of America; 4 Department of Biochemistry and Biophysics, Linus Pauling Institute, Oregon State University, Corvallis, Oregon, United States of America; 5 Cancer Science Institute of Singapore, National University of Singapore, Singapore, Singapore; Centre de Recherche Public de la Santé (CRP-Santé), Luxembourg

## Abstract

The CCAAT/enhancer binding proteins (C/EBPs) are transcription factors involved in hematopoietic cell development and induction of several inflammatory mediators. Here, we generated C/EBPβ and C/EBPε double-knockout (*bbee*) mice and compared their phenotypes to those of single deficient (*bbEE* and *BBee*) and wild-type (*BBEE*) mice. The *bbee* mice were highly susceptible to fatal infections and died within 2–3 months. Morphologically, their neutrophils were blocked at the myelocytes/metamyelocytes stage, and clonogenic assays of bone marrow cells indicated a significant decrease in the number of myeloid colonies of the *bbee* mice. In addition, the proportion of hematopoietic progenitor cells [Lin(−)Sca1(+)c-Kit(+)] in the bone marrow of the *bbee* mice was significantly increased, reflecting the defective differentiation of the myeloid compartment. Furthermore, microarray expression analysis of LPS- and IFNγ-activated bone marrow-derived macrophages from *bbee* compared to single knockout mice revealed decreased expression of essential immune response-related genes and networks, including some direct C/EBP-targets such as *Marco* and *Clec4e*. Overall, the phenotype of the *bbee* mice is distinct from either the *bbEE* or *BBee* mice, demonstrating that both transcription factors are crucial for the maturation of neutrophils and macrophages, as well as the innate immune system, and can at least in part compensate for each other in the single knockout mice.

## Introduction

Mature myeloid cells including monocytes/macrophages and granulocytes differentiate from common myeloid progenitors (CMP) which originate from hematopoietic stem cells [1], [2]. Monocytes/macrophages and granulocytes including basophils, eosinophils and neutrophils are involved in the innate immune system for host defense. These cells can phagocytose infectious agents and produce inflammatory-associated cytokines. Several murine knockout models revealed that development and differentiation of these cells are controlled by transcription factors; and one of the major regulators is the CCAAT enhancer binding protein (C/EBP) family. Members of this family play important roles for proliferation, differentiation and apoptosis in a variety of cell types [3]–[5]. Their amino end contains a transcriptional activation domain, and the carboxyl terminal region has a basic leucine zipper motif that forms homo- or hetero-dimers and allows binding to DNA.

C/EBPα plays a crucial role for granulopoiesis; and mice deficient for the *Cebpa* gene lack neutrophils and eosinophils, and accumulate immature myeloid cells [6], [7]. Inactivating mutations and/or gene silencing via methylation of the promoter region of the human *CEBPA* gene often occur in acute myeloid leukemia [8]–[11].

C/EBPβ expression is dramatically induced during macrophage differentiation [12], [13]; and macrophages from C/EBPβ knockout mice have a defective ability to kill bacteria and tumor cells [14]–[16]. Cytokines including IL-6, TNFα and G-CSF are abundantly produced in wild-type macrophages stimulated with mIFNγ and LPS, but their expression is diminished in C/EBPβ knockout macrophages [14], [17], [18]. Furthermore, C/EBPβ-deficient mice lack emergent neutrophil production in response to cytokines and/or infection [19]; and their neutrophils have an enhanced ability to undergo apoptosis [20], suggesting that C/EBPβ is essentially involved in the production and survival of neutrophils.

Unlike other family members, expression of C/EBPε is restricted to myeloid lineage cells and not detected in non-hematopoietic tissues and cells [21], [22]. Therefore, myelopoiesis is also regulated by C/EBPε, and its expression parallels granulocytic differentiation [21]. C/EBPε interacts with the cell cycle regulators, retinoblastoma and E2F1 during granulopoiesis and induces terminal differentiation of granulocytes [23]. Recently, we and others have shown that C/EBPε-deficient mice develop normally, but fail to generate functional neutrophils with decreased uptake of bacteria and low expression of secondary and tertiary granule proteins [22], [24], [25]. The phagocytic function of C/EBPε-deficient macrophages is also impaired, and macrophage-specific genes including CD14, MCP-3 and PAI-2 are down-regulated [26]. The human *CEBPE* gene produces 4 isoforms (32, 30, 27 and 14 kDa C/EBPε proteins), and function of these isoforms differs. The 32 and 30 kDa C/EBPε works as transcriptional activator, the 27 kDa protein as transcriptional repressor, and the 14 kDa form as dominant-negative regulator [27].

The structure of C/EBPβ and C/EBPε proteins is similar; especially the C-terminal regions of these two molecules with over 70% homology. Since expression of C/EBPβ and C/EBPε overlaps in the development of myeloid cells, both factors play crucial roles and may, at least in part, functionally compensate for each other in myelopoiesis and innate immune response.

In the present study, we generated C/EBPβ and C/EBPε double knockout mice and analyzed their hematopoietic system, as well as their inflammatory response. Compared to the single knockout and wild-type mice, the double knockout mice were highly susceptible to fatal infections, had morphologically immature neutrophils, lacked production of important host defense-related genes, and had an impaired proliferative activity of hematopoietic stem cells. Since this aberrant phenotype was not found in the single knockout mice, our findings indicate that both C/EBPβ and C/EBPε are required for the maturation of neutrophils and macrophages, as well as the innate immune system, and can at least in part compensate for each other in the single knockout genotype.

## Materials and Methods

### Cytokines, antibodies and other reagents

Murine granulocyte/macrophage-colony stimulating factor (GM-CSF), G-CSF, stem cell factor (SCF), interleukin-3 (IL-3), and human IL-6 were purchased from Calbiochem (San Diego, CA, USA). Human erythropoietin (EPO) was from Amgen (Thousand Oaks, CA, USA), murine macrophage stimulating factor (M-CSF) and interferon-γ (mIFNγ) from PeproTech, Inc. (Rocky Hill, NJ, USA), MethoCult media M3234 from StemCell Technologies (Vancouver, British Columbia, Canada), RPMI 1640 medium and phosphate-buffered saline (PBS) from Invitrogen (Carlsbad, CA, USA), and fetal bovine serum (FBS) from Atlanta Biologicals (Lawrenceville, GA, USA). Anti-phospho-STAT3 (Tyr705) and anti-STAT3 antibodies were obtained from Upstate Cell Signaling (Lake Placid, NY, USA) and Santa Cruz Biotechnology (Santa Cruz, CA, USA), respectively.

### Generation and genotyping of mice

Mice were bred under sterile conditions in the animal housing facility at Cedars-Sinai Medical Center, and all animal experiments were in accordance with the guidelines of Cedars-Sinai Research Center and the NIH (IACUC protocol number is 2292). Single knockout mice of either C/EBPβ (*bbEE*; C57bl/6) or C/EBPε (*BBee*; 129/SvEv x NIH Black Swiss) were generated as described previously [14], [22]. Heterozygous-deficient mice of both C/EBPβ and C/EBPε (*BbEe*) were generated by mating *bbEE* males and *BBee* females under pathogen-free conditions; and *BbEe* mice were intercrossed to produce homozygous-deficient mice for both genes (*bbee*).

Mouse tail-tips were digested in buffer containing 10 mM Tris-HCl (pH 8.0), 100 mM EDTA, 0.5% SDS and 0.1 mg/mL proteinase K (Sigma-Aldrich, St. Louis, MO, USA), overnight at 50°C. Genomic DNA was isolated by phenol/chloroform extraction followed by ethanol precipitation, and resuspended in 1 mL of TE buffer. To determine genotype of mice, 5 primers termed NF14 (5′- ATG CAA TCC GGA TCA AAC GTG GCT GAG-3′), NENF1823R (5′- CTT TAA TGC TCG AAA CGG AAA AGG TTC -3′), Neo1500 (5′- ATC GCC TTC TAT CGC CTT CTT GAC GAG -3′), mepsilon S (5′- GCT ACA ATC CCC TGC AGT ACC -3′) and, mepsilon AS (5′- TGC CTT CTT GCC CTT GTG -3′) were utilized. To detect each allele, the following combination of primers were used: NF14 and NENF 1823R for wild-type allele of the *Cebpb* gene, Neo1500 and NENF 1823R for knockout allele of the *Cebpb* gene, mepsilon S and mepsilon AS for wild-type allele of the *Cebpe* gene, and mepsilon S and Neo1500 for knockout allele of the *Cebpe* gene. Genomic PCR was performed using FailSafe PCR buffer of PreMix I for the *Cebpb* gene and PreMix F for the *Cebpe* gene (Epicentre Biotechnologies, Madison, WI, USA).

### Isolation of neutrophils and mononuclear bone marrow cells, and cultivation of macrophages

Neutrophils were collected by peritoneal lavage with ice-cold PBS at 18 h after intraperitoneal injection with 1.5 mL of 4% sterile thioglycollate (Sigma-Aldrich). After centrifugation, peritoneal cells were resuspended in RPMI 1640 supplemented with 10% FBS. To isolate non-adherent neutrophils, the lavage cells were cultured for 3 h, and non-adherent cells were collected. Cytospins were prepared and stained with Diff-Quick (Dade Behring, Dudingen, Switzerland).

Bone marrow cells were harvested from adult mice at 6–8 weeks of age. Mononuclear cells were isolated from bone marrow cells using Lymphocyte Separation Medium (Mediatech, Manassas, VA, USA). Mononuclear cells were cultured with 10 ng/mL mM-CSF in RPMI 1640 with 10% FBS for 7 days to induce macrophage differentiation. Non-adherent cells were removed, and morphological inspection showed that >98% of cells were macrophages. The bone marrow-derived macrophages were cultured either with or without 100 ng/mL LPS (Sigma-Aldrich) and 100 ng/mL mIFNγ.

### Microarray analysis of gene expression

Total RNA was extracted from the stimulated macrophages with the RNeasy Mini kit with on-column DNase digestion (Qiagen, Valencia, CA, USA), and 3–5 µg of high-quality RNA were converted, labeled and subsequently hybridized to the Mouse 430 2.0 oligonucleotide chips (Affymetrix, Santa Clara, CA, USA) according to the manufacturer's protocol. The microarray screens were conducted at the DNA Microarray facility of UCLA, Los Angeles. The probes were normalized with the MASv5.0 software for processing Affymetrix oligonucleotide array data, and genes were considered to be differentially expressed with a fold change level of ≤2 or ≥2, respectively. The Multi Experiment Viewer (MeV) v4.4.1 software was used to analyze the normalized expression data in clusters. Microarray data is MIAME compliant and the raw data has been deposited in the Gene Expression Omnibus (GEO) database, number GSE23821.

### Signaling pathway analysis

To set up a potential network based on the regulated genes to identify the molecular events and to generate a signaling network based on the known interaction/relations among target genes/proteins, we applied a reliable bioinformatics approach, Ingenuity Pathways Analysis (IPA, Ingenuity Systems, http://www.ingenuity.com, Mountain View, CA, USA). The software ranks networks by a score that takes into account the number of focus genes and the size of the networks, indicating the likelihood of the focus genes in a network being found together by chance. The higher the score (score  = −log(p-value) from Fisher's exact test analysis), the lower is the probability of finding the observed Network Eligible Molecules in a given network by chance. Genes in our microarray data set that were collectively deregulated in the comparison of *BBEE*, *bbEE*, and/or *BBee* with *bbee* were considered for the pathway analysis.

### RT-PCR, analysis of peripheral blood cell counts and chromatin immunoprecipitation (ChIP) assay

Total RNA was isolated from bone marrow cells and bone marrow-derived macrophages using RNeasy Mini Kit (Qiagen). Total RNA (1 µg) was converted into cDNA by reverse transcription with Superscript III (Invitrogen). Gene expression was quantified with real-time quantitative PCR (iCycler, Bio-Rad, Hercules, CA, USA) using Sybr Green; and expression levels of target genes were normalized with β-actin.

Peripheral blood samples were obtained from the ocular sinus and counted using a HEMAVET 850 (Drew Scientific Inc., Dallas, TX, USA).

For chromatin immunoprecipitation assay, ChIP assay kit (Upstate Biotechnology, Lake Placid, NY) was used, and chromatin was prepared for IP as instructed by the manufacturer protocol. The sonicated chromatin was immunoprecipitated with either 5 µg of anti-C/EBPβ or anti-C/EBPε antibodies (Santa Cruz Biotechnology) or normal rabbit IgG antibody as negative control (Upstate Biotechnology). Immunoprecipitated DNA was subsequently analyzed by PCR using primers specific for the promoter region of the respective target genes (the *Marco* and *Clec4e* genes). C/EBP binding sites were elucidated by using a web-based search tool on http://www.cbrc.jp/research/db/TFSEARCH.html. Input chromatin was analyzed for β-actin as a positive control. PCR products were analyzed by 2.5% agarose/ethidium bromide gel electrophoresis. All primer sequences are shown in Table 1.

**Table 1:** Primer sequences.

For PCR analysis	
G-CSFR F	GGGTCCACCAACAGTACAGG
G-CSFR R	CAACCAGGAGCTCAGGCTAC
β-actin F	CCTGAGGAGCACCCTGTG
β-actin R	ATCACAATGCCTGTGGTACG
MRC1 F	TGGAGAGCTGGCGAGCATCAAG
MRC1 R	ACCATCACTCCAGGTGAACCCCTC
MARCO F	CCACCTGATCCTGCTCACGGC
MARCO R	GCCCACTGCAGCGAGAAGAAGG
CLEC4E F	GTGGAGGGTCAGTGGCAATGGG
CLEC4E R	GGTGGCACAGTCCTCCACCA
SOCS2 F	TCAGCTGGACCGACTAACCT
SOCS2 R	CAGGTGAACAGTCCCATTCC
CD38 F	TCTTCCTGGGACGCTGCCTCA
CD38 R	AGTGGGGCGTAGTCTTCTCTTGT
TNFα F	ATCCGCGACGTGGAACTGGC
TNFα R	TGAGTGTGAGGGTCTGGGCCAT

For ChIP analysis	
MARCO promoter F	TGCCACAGGTTCAACAGGGA
MARCO promoter R	TGGGTTCAAATCTCCAGACC
CLEC4E promoter F	TCTGGCTCATCAAACACGGGGA
CLEC4E promoter R	CTCTGCCCACACCACACAGC
β-actin F	ACCTGTTACTTTGGGAGTGGCAAGC
β-actin R	GTCGTCCCAGTTGGTAACAATGCC

### Colony assay and Western blot analysis

Colony assays were performed as described previously [20]. Briefly, mononuclear bone marrow cells (2×10^4^) were cultured in 1% methylcellulose (Stemcell Technologies, Vancouver, BC, Canada) supplemented with either IL-3 (10 ng/mL), IL-6 (10 ng/mL), SCF (20 ng/mL), and EPO (3 U/mL); or GM-CSF (20 ng/mL); or G-CSF (60 ng/mL); or G-CSF (60 ng/mL) and SCF (20 ng/mL).

For Western blot detection of phospho-STAT3, bone marrow cells were cultured either with or without 100 ng/mL of G-CSF for 30 min. These samples were subjected to SDS-PAGE followed by an electro-transfer to polyvinylidene difluoride membranes which subsequently were probed with anti-phospho-STAT3 or anti-STAT3 antibodies. The signals were developed with Supersignal West Pico-Chemiluminescent (Pierce Biotechnology, Rockford, IL, USA).

### FACS analysis of bone marrow cells

Fc receptors were blocked using purified rat anti-mouse CD16/CD32 monoclonal antibodies (BD Pharmingen, Franklin Lakes, NJ, USA). Cells were then stained using rat anti-mouse antibodies (BD Pharmingen) at concentrations recommended by the manufacturer. Analysis of the primitive hematopoietic cell compartment containing the c-Kit^+^/Sca-1^+^/Lineage^−^ (KSL) fraction was carried out using a combination of APC mouse Lineage antibody cocktail (to deplete cells expressing hematopoietic lineage markers), CD117 (c-kit) FITC and Sca-1 PE. Cells were incubated with FACS antibodies for 15 minutes at 4°C and washed once with PBS before analysis on a FACS Calibur (BD Biosciences). Compensation was performed using BD CompBeads Anti-Rat Ig, κ. FACS analysis was performed using the Flo-Jo program (Tree Star Inc, Palo Alto, CA).

## Results

### Generation of double knockout (*bbee*) mice

Heterozygous-deficient mice [C/EBPβ and C/EBPε (*BbEe*)] were generated by mating C/EBPβ^−/−^ (*bbEE*) males and C/EBPε^−/−^ (*BBee*) females, since *bbEE* female mice are sterile [28]. Genotypes of all newborn mice were *BbEe*; and they appeared normal (data not shown). These mice were intercrossed to produce homozygous-deficient mice for both genes (*bbee*). As shown in Table 2, all allele frequencies were in a manner following Mendelian law; and 7 out of 146 mice (4.79%) were *bbee* mice including 4 females and 3 males.

**Table 2:** Genotype and number of newborns from *BbEe* intercrosses.

Genotype	Number of newborns	Ratio
*BBEE*	10	6.85%
*BbEE*	19	13.01%
*bbEE*	3	2.05%
*BBEe*	28	19.18%
*BBee*	8	5.48%
*BbEe*	36	24.66%
*Bbee*	22	15.07%
*bbEe*	13	8.90%
*bbee*	7	4.79%
	146	100%

*BbEe* mice were intercrossed to produce homozygous-deficient mice for both genes (*bbee*). All allele frequencies were in a manner following Mendelian law.

**Figure 1:**
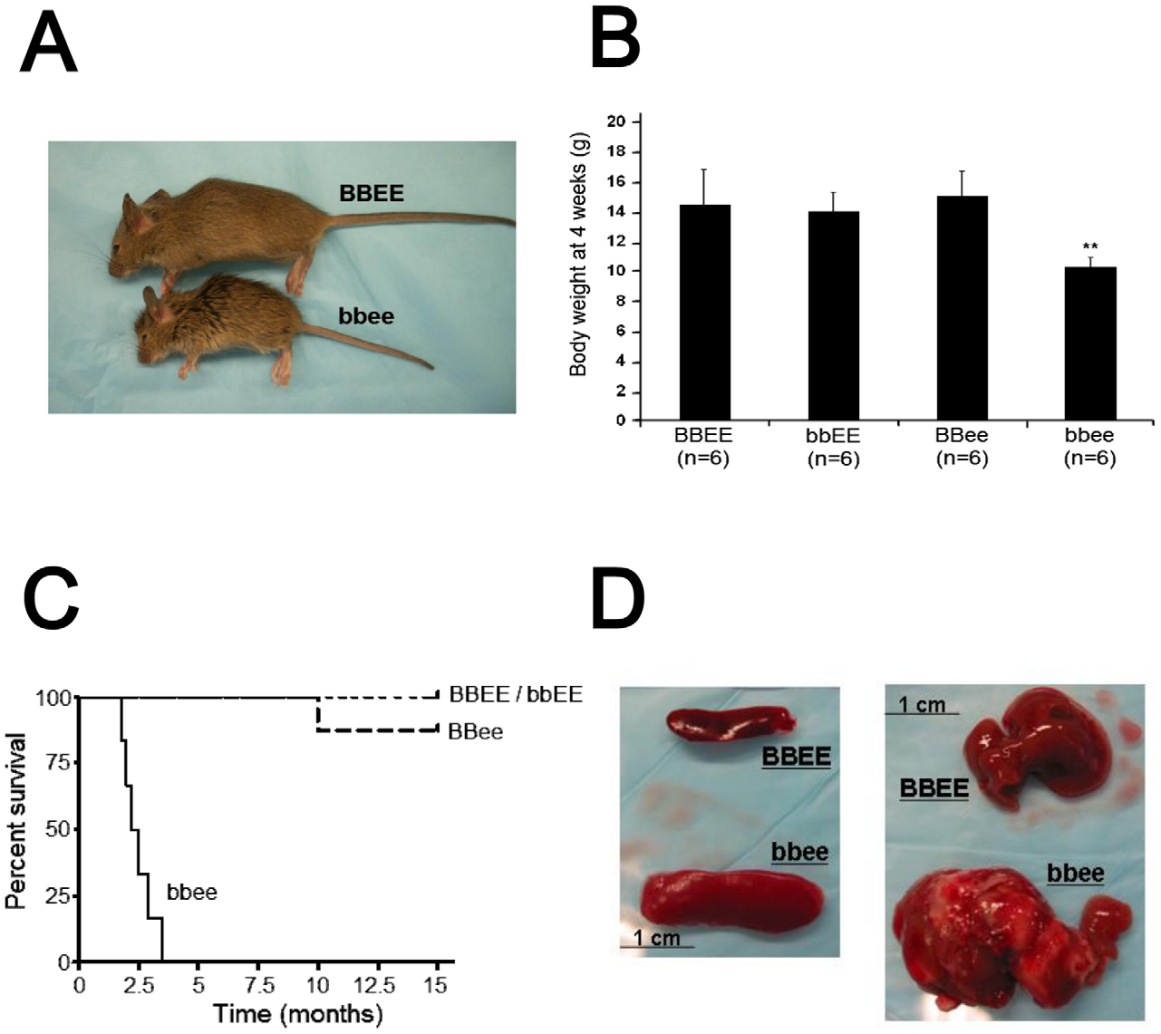
C/EBPβ^−/−^C/EBPε^−/−^ mice are highly susceptible to fatal infections. (A) Comparison between wild-type (*BBEE*) and C/EBPβ^−/−^C/EBPε^−/−^ (*bbee*) mice (both at 4 weeks of age). The *bbee* mice were smaller than *BBEE* mice and had rougher hair. (B) Body weight of four genotypic subtypes of mice (n = 3 per genotype). Body weights of *bbEE* and *BBee* mice were comparable to *BBEE* mice; but *bbee* mice weighed less than the other subtypes of mice at 4 weeks after birth (*p* = 0.05, *t*-test). (C) Kaplan Meier survival curves of four genotypic subtypes of mice. The *bbee* mice died 2–3 months after birth due to fatal bacterial infections. (D) Enlarged spleen and abscess formation in the liver in the *bbee* mice. Spleen size (left panel) was increased in the *bbee* mice (2 months). Liver (right panel) had visible abscesses (2 months).

Size of *bbee* mice were smaller than wild-type (*BBEE*) mice; and their hair was rough (Figure 1A). Body weight of *bbee* mice at 4 weeks after birth was 11.75±1.04 g (mean ± S.D.), whereas, *BBEE* mice were 13.25±0.5 g (*p* = 0.05, *t*-test; Figure 1B). Morphological features observed by histochemical analysis showed that all tissues examined including lung, liver, pancreas, spleen, heart, kidney and testis of *bbee* mice were normally developed (data not shown). Furthermore, a *bbee* male was mated with a *Bbee* female; and 2 of 7 newborns were *bbee* mice (data not shown), showing that *bbee* males are fertile. Taken together, these results indicate that embryogenesis, early development, as well as organogenesis including male reproduction of *bbee* mice were normal.

### Susceptibility to infections of double knockout (*bbee*) mice

Analysis of peripheral blood cell counts revealed that the numbers of white blood cells, neutrophils, red blood cells and platelets were almost the same among *BBEE*, *bbEE*, *BBee* and *bbee* mice (Table 3). Lymphocyte counts in *BBee* and *bbee* mice were lower than in the two other genotypes, but the difference was not significant. Only the number of monocytes was significantly higher in *BBee* and *bbee* mice compared to *BBEE* and *bbEE*.

**Table 3:** Blood cell counts in *BBEE*, *bbEE*, *BBee*, and *bbee* mice.

Cell types		*BBEE* (n = 3)	*bbEE* (n = 4)	*BBee* (n = 3)	*bbee* (n = 4)
White blood cells	(K/uL)	15.84±3.22	13.9±3.39	8.85±2.66	10.995±2.74
Neutrophils	(K/uL)	1.85±1.33	2.395±0.55	1.53±0.38	2.06±0.93
Lymphocytes	(K/uL)	13.52±1.91	11.1175±2.93	6.33±2.08	8.11±2.03
Monocytes	(K/uL)	0.45±0.03	0.3475±0.04	0.9±0.22*	0.76±0.21*
Red blood cells	(M/uL)	8.955±2.2	9.505±0.7	7.50±1.15	8.27±0.9
Platelets	(K/uL)	610±492.15	424±27.02	452±67.52	436±56.68

Blood samples from mice of each genotype were analyzed (K, ×1000). Values represent the mean ± SD.

*Number of monocytes was significantly higher in *BBee* and *bbee* mice compared with *BBEE* mice (*p*<0.05, *t*-test).

Interestingly, *bbee* mice died between 2–3 months of age (Figure 1C). These mice had enlarged spleens (Figure 1D, left panel) and suffered from systemic infections with abscess formation in their livers (Figure 1D, right panel), lungs and peritoneum (data not shown). *In vitro* culture of visible hepatic, peritoneal and lung abscesses revealed infections with *Streptococcus viridians*, *Enterococcus species* and *Escherichia coli* in the *bbee* mice (data not shown). Mice with *bbEE* and *BBee* as a genotype showed no signs of infection during life or at autopsy, with survivals equivalent to *BBEE* mice. These results demonstrated that *bbee* mice were highly susceptible to bacterial infections.

### Block of morphological differentiation of neutrophils in *bbee* mice

**Figure 2:**
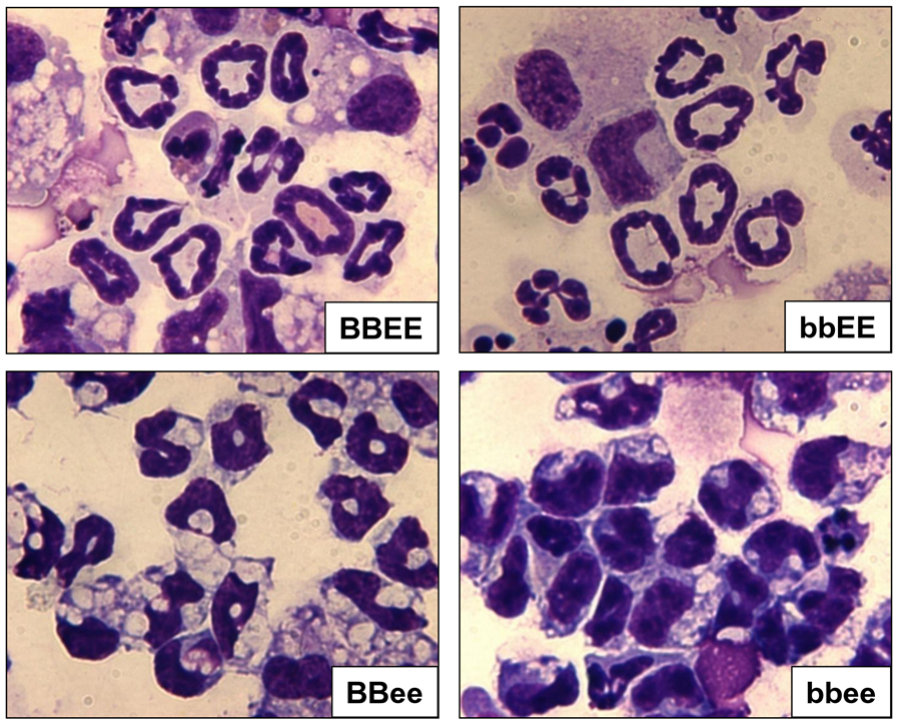
Morphological characterization of neutrophils harvested by lavage from peritoneal cavity. Morphological characterization of neutrophils harvested by lavage from peritoneal cavity. Wild-type, C/EBPβ^−/−^ (*bbEE*), C/EBPε^−/−^ (*BBee*) and C/EBPβ^−/−^C/EBPε^−/−^ (*bbee*) mice were injected with 1.5 mL of 4% thioglycollate into their peritoneal cavity. After 18 h, neutrophils were harvested by lavage. Cytopreps of cells were stained with Diff-Quick.

Next, we examined the morphology of the neutrophils as one of the major contributors of host defense. Neutrophils harvested from the thioglycollate stimulated peritoneal cavity of *BBEE* and *bbEE* mice were morphologically mature with segmented nuclei (Figure 2). In *BBee* mice, terminal differentiation of neutrophils was incomplete with atypical bi-lobed nuclei. Notably, the *bbee* mice showed a highly defective differentiation of neutrophils with large and in part indented nuclei (Figure 2), representing a potential block at the myelocytes to metamyelocytes stage of differentiation.

### Impaired response to cytokines and accumulation of undifferentiated hematopoietic progenitor cells in *bbee* bone marrow

**Figure 3:**
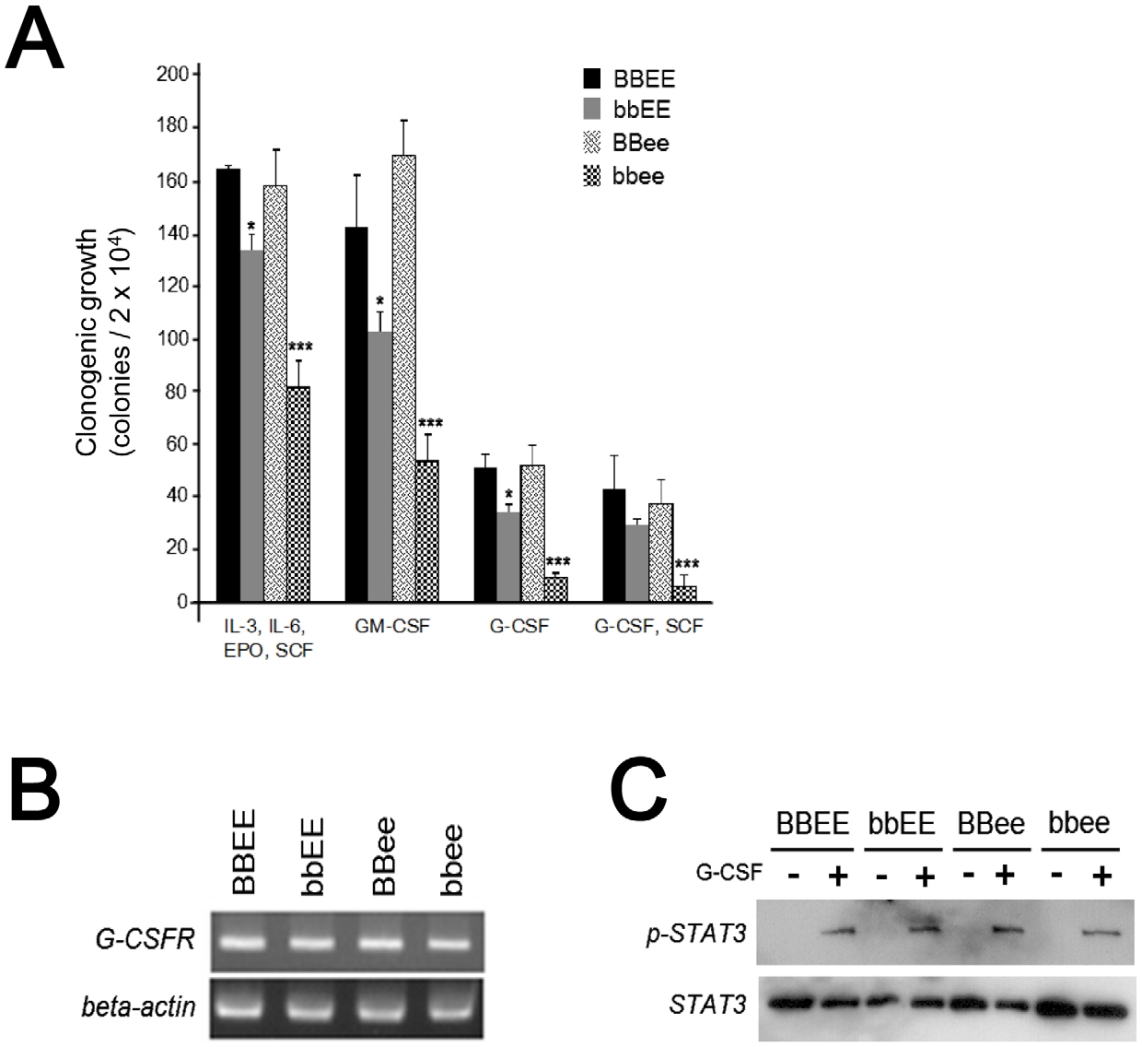
Characterization of hematopoietic cells in C/EBPβ^−/−^C/EBPε^−/−^ mice. (A) Colony assay in methylcellulose. Light-density, mononuclear bone marrow cells (2×10^4^) were cultured in 1% methylcellulose supplemented with either IL-3 (10 ng/mL), IL-6 (10 ng/mL), SCF (20 ng/mL), and EPO (3 U/mL); or GM-CSF (20 ng/mL); or G-CSF (60 ng/mL); or G-CSF (60 ng/mL) and SCF (20 ng/mL). Colonies were scored if they contained ≥50 cells at day 7 of culture. Results represent the mean ± SD of 3 mice. (B) Expression of G-CSF receptor (G-CSFR) in mononuclear bone marrow cells. Expression of G-CSFR mRNA was examined by RT-PCR. β-actin was used as an internal control. (C) Expression of STAT3 in mononuclear bone marrow cells. The cells were stimulated either with or without G-CSF (100 ng/mL) for 30 min. Levels of phospho-STAT3 (pSTAT3) and STAT3 were detected by Western blot analysis.

Next, we examined hematopoietic progenitor cells to assess whether they respond to cytokines and produce mature cells *in vitro*. Bone marrow cells from the *bbee* mice, as well as the other 3 type of mice, were cultured with either combinations of four cytokines (IL-3, IL-6, EPO and SCF), GM-CSF, G-CSF, or combination of two cytokines (G-CSF and SCF). Interestingly, the number of clonogenic cells of the *bbee* mice was markedly diminished compared to clonogenic growth of wild-type, *bbEE* and *BBee* bone marrow cells (Figure 3A).

The level of G-CSF receptor mRNA in the bone marrow cells of the *bbee* mice was similar to levels present in the other three types of mice (Figure 3B). G-CSF stimulation is known to induce activation of STAT3 via stimulation of the G-CSF receptor. Phospho-STAT3 was detected in the G-CSF-stimulated bone marrow cells at comparable levels in all four types of mice (Figure 3C). Taken together, these results indicate that *bbee* hematopoietic progenitor cells have an impaired ability to form colonies, but the secondary signals stimulated by G-CSF appeared to be intact in these cells.

**Figure 4:**
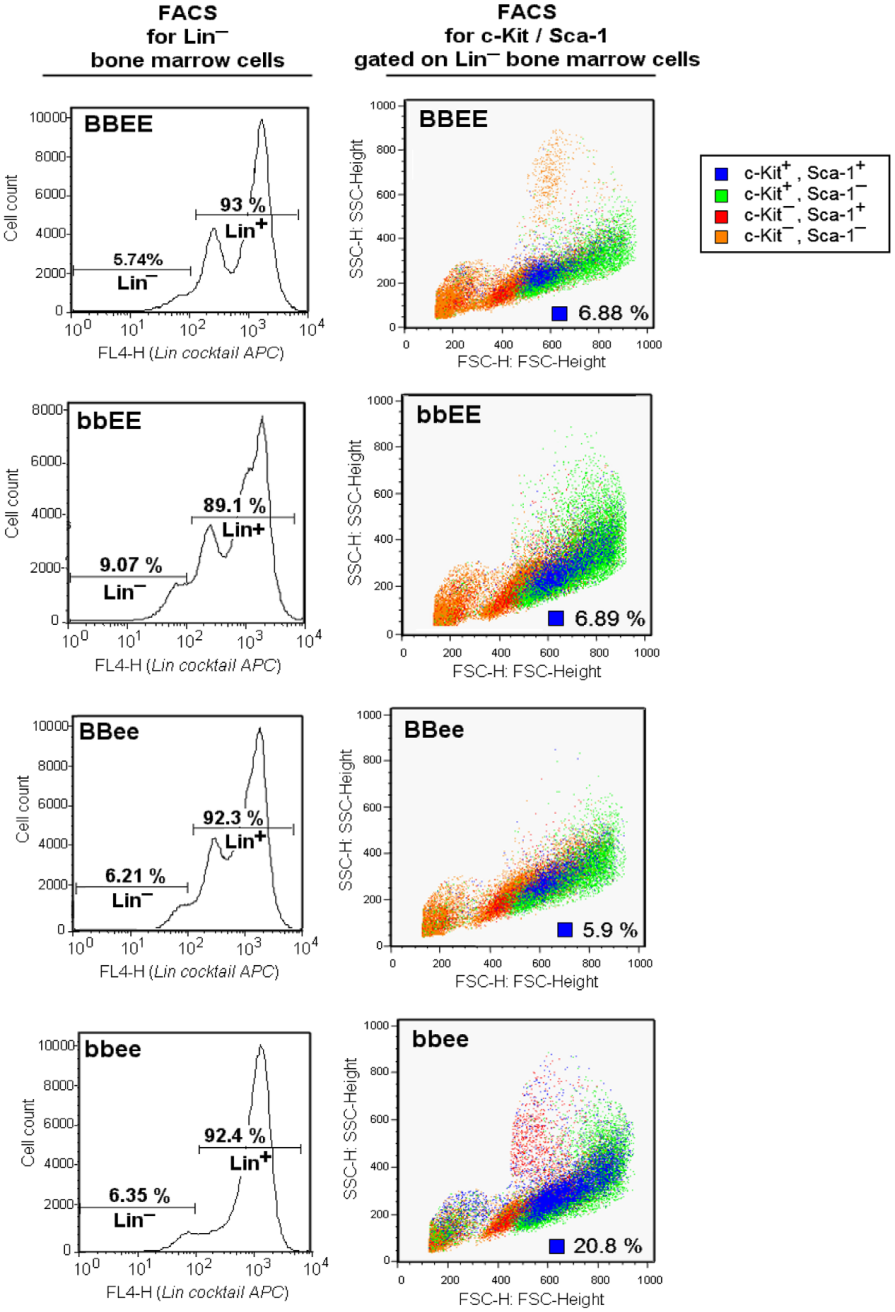
Ratio of hematopoietic progenitor cells. The lineage-negative fraction of bone marrow cells was sorted with FITC-conjugated anti-c-Kit and PE-conjugated anti-Sca-1 antibodies. The hematopoietic progenitor cell population [KSL population (c-Kit^+^, Sca-1^+^, and Lineage^−^; blue dots)] was compared between the wild-type (*BBEE*), C/EBPβ^−/−^ (*bbEE*), C/EBPε^−/−^ (*BBee*) and C/EBPβ^−/−^C/EBPε^−/−^ (*bbee*) mice.

The primitive hematopoietic cell compartment containing hematopoietic stem cells is known as the c-Kit^+^/Sca-1^+^/Lineage^−^ (KSL) fraction. FACS analysis demonstrated that *BBEE*, *bbEE* and *BBee* bone marrow cells contained 5–7% c-Kit^+^/Sca-1^+^ cells in the Lineage negative fraction (Figure 4). In contrast, *bbee* bone marrow cells had a markedly increased cell population in this fraction (20.8%), suggesting that a block of hematopoietic differentiation leads to accumulation of KLS cells in the bone marrow of *bbee* mice.

### Dysregulated gene expression of bone marrow-derived macrophages from *bbee* mice

**Figure 5:**
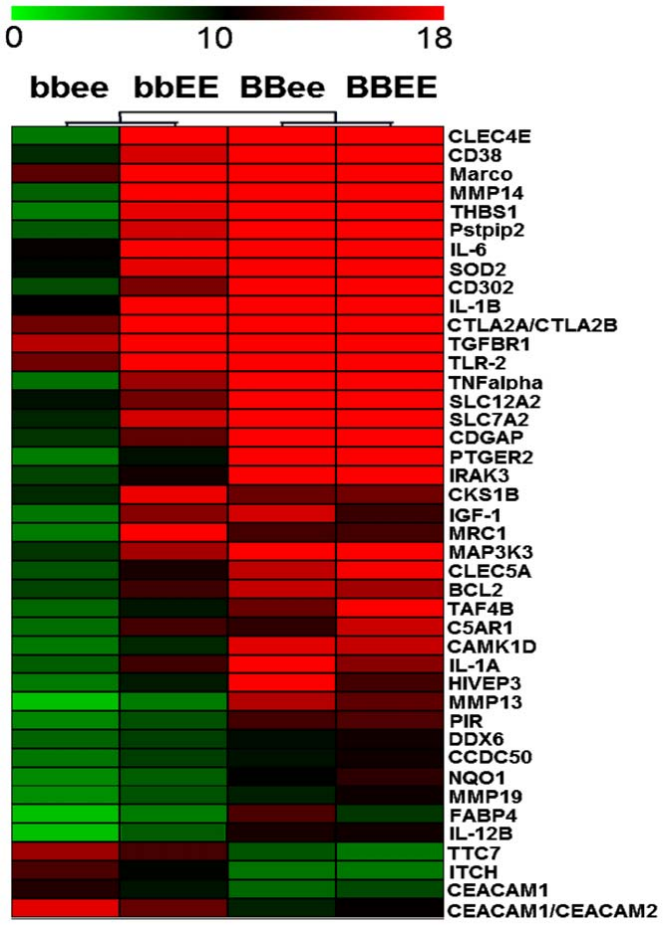
Cluster analysis of microarray data of bone marrow derived macrophages stimulated with LPS and mIFNγ. Expression data established from activated macrophages revealed a total of 145 genes that were collectively deregulated in the comparison of cells from either wild-type (*BBEE*), C/EBPβ^−/−^ (*bbEE)*, or C/EBPε^−/−^ (*BBee*) with C/EBPβ^−/−^C/EBPε^−/−^ (*bbee*). Cluster analysis performed with Multi Experiment Viewer (MeV) v4.4.1 indicates 45 of the most deregulated genes out of the collective of 145 genes.

Additionally, we analyzed the gene expression profile of bone marrow-derived macrophages to explore which inflammatory mediators were aberrantly regulated in the *bbee* mice compared to single deficient mice. Using microarray analysis, we detected a total of 145 genes that were collectively deregulated in the comparison of cells from either *BBEE*, *bbEE*, or *BBee* with *bbee*. A cluster analysis represents the 45 most deregulated genes out of the collective of the 145 genes (Figure 5). These included immune response-related genes, as well as proinflammatory cytokines including *Tnfα*, and *Il-6*.

**Figure 6:**
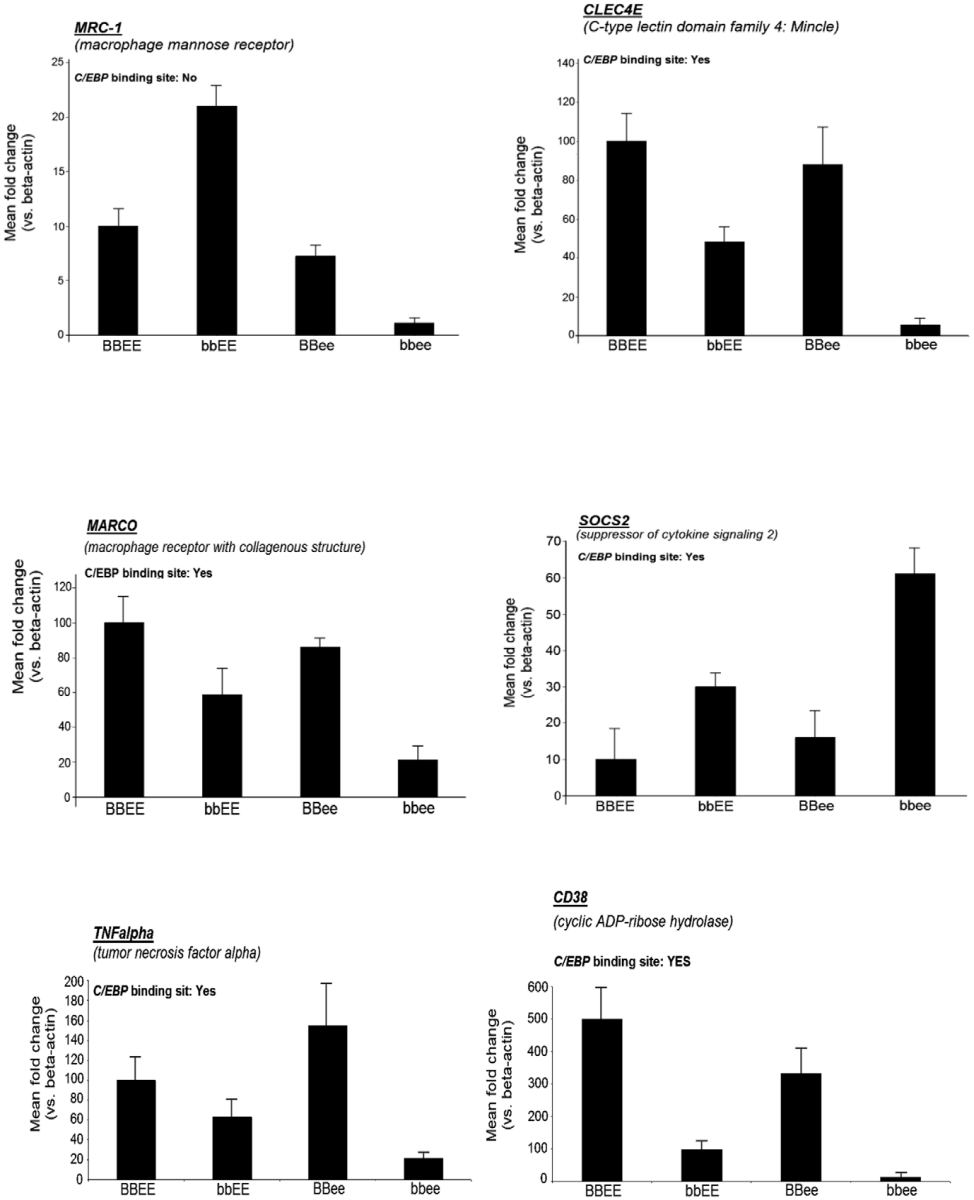
Validation of mRNA expression of *Mrc1*, *Clec4e*, *Marco*, *Socs2*, *Tnfα*, and *Cd38* in bone marrow-derived macrophages stimulated with LPS and mIFNγ. Expression levels of macrophage mannose receptor 1 (*Mrc1*), C-type lectin domain family 4, member e (*Clec4e*), macrophage receptor with collagenous structure (*Marco*), suppressor of cytokine signaling 2 (*Socs2*), *Tnfα* and *Cd38* mRNA in bone marrow derived macrophages stimulated with LPS and mIFNγ were determined by quantitative RT-PCR. C/EBP-binding site in the promoter region of these genes are also indicated.

Expression levels of macrophage mannose receptor 1 (*Mrc1*), C-type lectin domain family 4, member e (*Clec4e*), macrophage receptor with collagenous structure (*Marco*), suppressor of cytokine signaling 2 (*Socs2*), *Tnfα* and *Cd38* mRNA in activated bone marrow derived macrophages were also determined by quantitative RT-PCR. As shown in Figure 6, and consistent with our microarray data, *Mrc1*, *Clec4e*, *Marco*, *Tnfα* and *Cd38* were clearly downregulated, and *Socs2* was upregulated in *bbee* compared to *bbEE* or *Bbee* macrophages.

**Figure 7:**
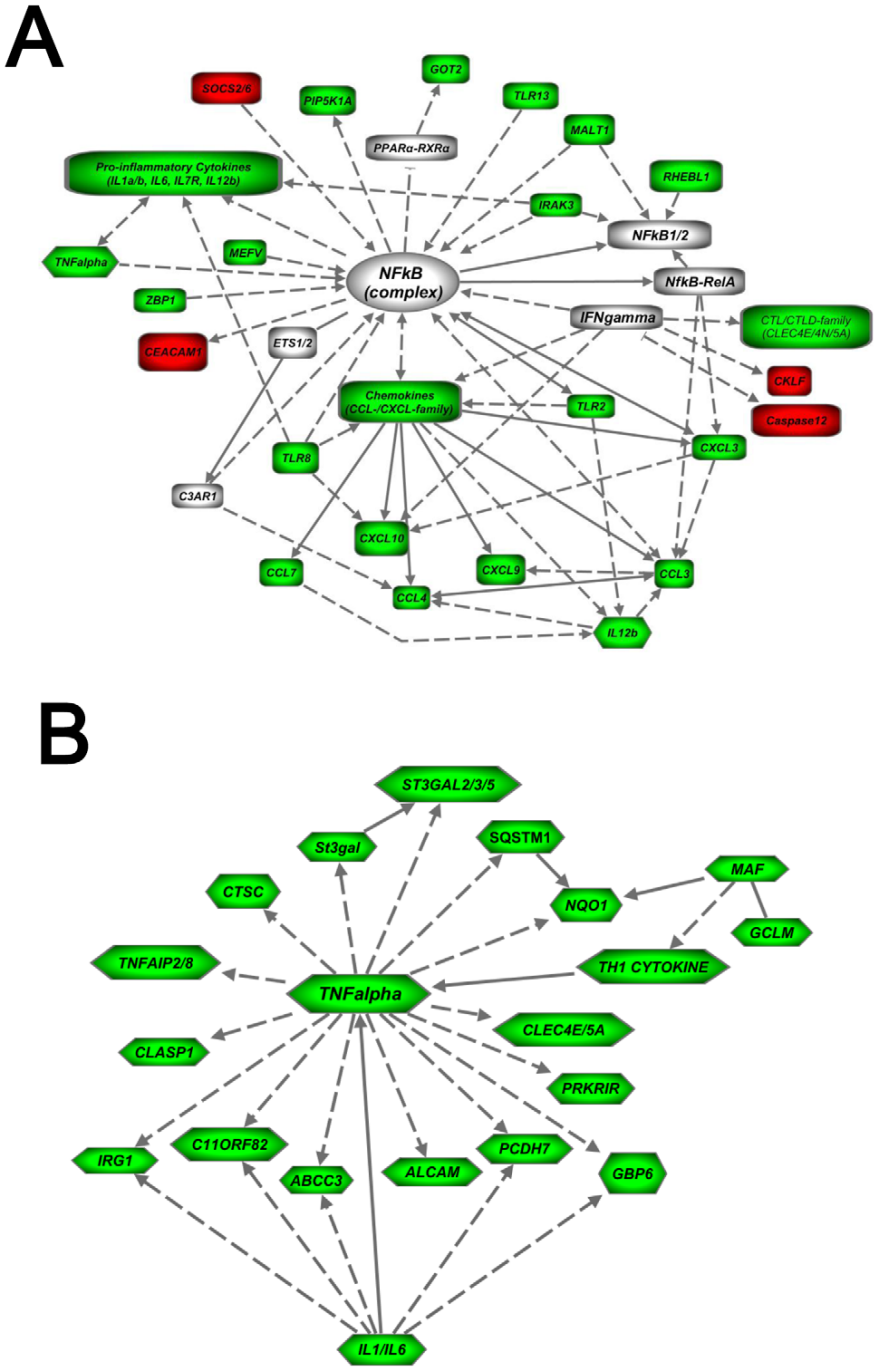
Pathway-based analysis. (A, B) Microarray expression analysis established from activated macrophages resulted in 2 pathways which were significantly (p<0.001) involved in the comparison of wild-type (*BBEE*), C/EBPβ^−/−^ (*bbEE)*, and/or C/EBPε^−/−^ (*BBee*) with C/EBPβ^−/−^C/EBPε^−/−^ (*bbee*). They both represent inflammatory response pathways involving pro-inflammatory cytokines and chemokines, as well as Toll-like receptor signaling molecules. Solid lines denote direct interactions, while dotted lines represent indirect interactions of genes/proteins. *Green*: downregulated (≤2 fold); *Red*: upregulated (≥2 fold) molecules.

Furthermore, we used the IPA software to assess the molecular pathways and their respective biological functions of the deregulated genes in the macrophages of the *bbee* mice compared to single deficient mice. Ingenuity analysis identified 10 pathways, whereas two met statistical and biological relevance for our study. Both pathways had statistical scores of >30, and unique gene clusters related to innate immunity and either TNFα or NFkB signaling, respectively (Figure 7). Taken together, these results show that activated macrophages of *bbee* mice have aberrant gene expression of some essential proinflammatory cytokines and networks, suggesting that both C/EBPβ and C/EBPε are critically involved in their regulation in macrophages.

### C/EBPβ and/or C/EBPε bind to the promoter region of the *Marco* and *Clec4e* genes

**Figure 8:**
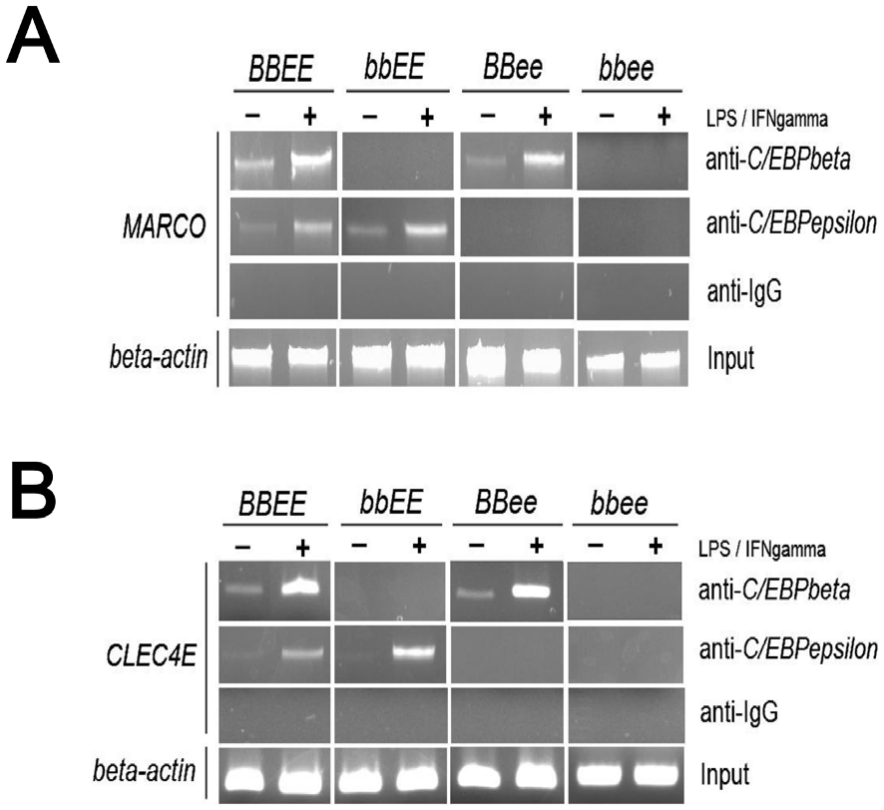
Chromatin immunoprecipitation (ChIP) assay in bone marrow derived macrophages stimulated with LPS and mIFNγ. Bone marrow derived macrophages stimulated with LPS and mIFNγ were subjected to ChIP assay by using anti-C/EBPβ, anti-C/EBPε or normal IgG antibodies. C/EBP-binding region of the *Marco* (A) and *Clec4e* (B) genes were amplified by specific primers.

Since the promoter regions of the *Marco*, *Clec4e*, *Socs2*, *Tnfa* and *Cd38* genes contain putative C/EBP-binding sites (Figure 6), we examined the binding of C/EBPβ and C/EBPε to the promoter regions of the *Marco* and *Clec4e* genes in macrophages. Chromatin immunoprecipitation assay revealed that both C/EBPβ and C/EBPε can bind to a C/EBP-binding site of either *Marco* or *Clec4e* in wild-type macrophages (Figure 8). The binding of the two C/EBP transcription factors was enhanced after stimulation of the macrophages with LPS and mIFNγ (Figure 8). Furthermore, C/EBPε bound to these promoter regions in *bbEE* macrophages, and C/EBPβ interacted with these sites in *BBee* macrophages, suggesting that C/EBPβ and C/EBPε can compensate for each other in single knockout macrophages.

## Discussion

Myelopoiesis is highly regulated by transcription factors including C/EBPα, C/EBPβ and C/EBPε. These transcription factors play important roles in myelopoiesis as highlighted by the studies of single knockout murine models [13], [29], [30], providing several unexpected findings. For example, C/EBPα-deficient mice, which normally do not have neutrophils, can produce them in the presence of a high concentration of cytokines with rapid upregulation of C/EBPβ in myeloid progenitors. Also, C/EBPβ-deficient mice are unable to produce “emergency” neutrophils when stimulated by either cytokines or infection, suggesting that C/EBPβ is important for the production of these neutrophils [19], [20].

C/EBP family proteins share a high degree of amino acid sequence similarity in their dimerization and DNA-binding domains, and potentially bind the same cis-elements in promoters [3], [4]. This may allow for one family member to compensate for the loss of another. For example, we previously showed that C/EBPα-deficient mice expressing C/EBPβ from the *Cebpa* gene locus had normal hematopoiesis, indicating that C/EBPβ can substitute for functions of C/EBPα in hematopoietic cells [31]. C/EBPα-deficient mice have a severe loss of liver function; in contrast, the C/EBPα-deficient mice expressing C/EBPβ from the *Cebpa* locus displayed normal liver function [32]. Double knockout mice of C/EBPα and C/EBPβ die between E10 and E11 and have a defective placenta; contrary, single knockout mice of C/EBPα and C/EBPβ develop normally at that embryonic stage [33]. In addition, the activities of C/EBPα, C/EBPβ and C/EBPδ are redundant in LPS-induced expression of IL-6 and monocyte chemoattractant protein-1 [34].

C/EBPε is involved in the functional maturation of neutrophils and macrophages both of which represent major elements of the innate immune system. A recent study showed that C/EBPβ and C/EBPε regulate the expression of cytokines including IL-8 in human neutrophils [35], demonstrating that C/EBP proteins play important roles for induction of cytokine expression in neutrophils. Importantly, a highly immature state of neutrophils was detected in the *bbee* mice with a potential differentiation arrest at the myelocytes/metamyelocytes stage. Taken together, C/EBPβ and C/EBPε are required not only for cytokine expression, but also for the terminal differentiation of the neutrophil.

Our colony formation assay revealed that *bbee* mononuclear bone marrow cells formed decreased numbers of myeloid colonies compared with the single knockout and wild-type mice in the presence of various cytokines including G-CSF, although expression of G-CSF receptor mRNA in the *bbee* cells was intact. Regulation of G-CSF receptor expression is controlled by C/EBPα [36], and *bbee* bone marrow cells expressed C/EBPα (data not shown). In addition, STAT3 activation, which is one of the downstream signals of G-CSF, also appeared to be intact, since the phosphorylation status of STAT3 stimulated with G-CSF was comparable among all 4 types of mice. The molecular mechanism of the impaired growth response to cytokines including G-CSF remains to be explored.

A slightly impaired response to cytokines was also found in *bbEE* bone marrow cells (current study and references 19 and 20), but *bbee* bone marrow cells showed a severe lack of response. One possible explanation for this finding is obtained from our hematopoietic cell fraction (KSL) analysis. KSL cells dramatically accumulated in the bone marrow of *bbee* mice. We assume that the accumulation is in part the result of impaired responses to differentiation-related cytokines. Therefore, these data strongly suggested that C/EBPβ and C/EBPε are required for commitment of hematopoietic progenitor cells under cytokine stimulation to myeloid cells.

C/EBPβ is dramatically induced during macrophage differentiation [12], [13]. C/EBPβ-deficient macrophages have a defect in their ability to kill bacteria [14]–[16], and the C/EBPβ isoform 34 kDa protein is responsible for induction of inflammatory cytokines in macrophages [37]. Expression of C/EBPε is upregulated during granulocyte differentiation, but not macrophage differentiation [21]; however, macrophages from C/EBPε-deficient mice have functional abnormalities [26]. Importantly, our current study revealed that the expression of several important proinflammatory cytokines and immune mediators is significantly repressed in activated bone marrow-derived macrophages from *bbee* mice as compared to single deficient mice. Some promoter regions of proinflammatory cytokine genes such as *Marco* and *Clec4e* contain C/EBP binding sites, and both C/EBPβ and C/EBPε can bind and regulate their expression. In single knockout mice, either C/EBPβ or C/EBPε might compensate for the loss of the other transcription factor allowing expression of the target gene. In contrast, *bbee* mice have neither transcriptional activators, C/EBPβ and C/EBPε, resulting in highly dysregulated gene expression in their macrophages.

In summary, the findings from the present study demonstrated that in contrast to the single knockout mice, synergistic effects of the absence of both genes were found in the double knockout mice. Severe aberrations involving the hematopoietic system and the innate immune response were present in the double knockout mice that were found either as a comparatively mild abnormality or even normal in the single knockout mice, indicating that C/EBPβ and C/EBPε can in part substitute for each other in the *BBee* or *bbEE* mice, respectively.
